# Trichomoniasis - are we giving the deserved attention to the most common non-viral sexually transmitted disease worldwide?

**DOI:** 10.15698/mic2016.09.526

**Published:** 2016-06-27

**Authors:** Camila Braz Menezes, Amanda Piccoli Frasson, Tiana Tasca

**Affiliations:** 1 Laboratório de Pesquisa em Parasitologia, Faculdade de Farmácia, Universidade Federal do Rio Grande do Sul. Porto Alegre, Rio Grande do Sul, Brazil.

**Keywords:** Trichomonas vaginalis, trichomoniasis, sexually transmitted disease, pathogenicity, MTZ

## Abstract

Etiology: *Trichomonas vaginalis* is the etiologic agent of trichomoniasis, the most common non-viral sexually transmitted disease (STD) in the world. Transmission: Trichomoniasis is transmitted by sexual intercourse and transmission via fomites is rare. Epidemiology, incidence and prevalence: The WHO estimates an incidence of 276 million new cases each year and prevalence of 187 million of infected individuals. However, the infection is not notifiable. Pathology/Symptomatology: The *T. vaginalis* infection results in a variety of clinical manifestations - in most cases the patients are asymptomatic, but some may develop signs typically associated to the disease. Importantly, the main issue concerning trichomoniasis is its relationship with serious health consequences such as cancer, adverse pregnancy outcomes, infertility, and HIV acquisition. Molecular mechanisms of infection: To achieve success in parasitism trichomonads develop a complex process against the host cells that includes dependent- and independent-contact mechanisms. This multifactorial pathogenesis includes molecules such as soluble factors, secreted proteinases, adhesins, lipophosphoglycan that culminate in cytoadherence and cytotoxicity against the host cells. Treatment and curability: The treatment with metronidazole or tinidazole is recommended; however, cure failures remain problematic due to noncompliance, reinfection and/or lack of treatment of sexual partners, inaccurate diagnosis, or drug resistance. Therefore, new therapeutic alternatives are urgently needed. Protection: Strategies for protection including sexual behavior, condom usage, and therapy have not contributed to the decrease on disease prevalence, pointing to the need for innovative approaches. Vaccine development has been hampered by the lack of long-lasting humoral immunity associated to the absence of good animal models.

## INTRODUCTION

The flagellate parasitic protozoan *Trichomonas vaginalis* was firstly described by Alfred François Donné in 1836 from a vaginal discharge. Although the infection has been considered as mild and curable sexually transmitted disease (STD), the high incidence/prevalence and increasing resistance to the treatment, as well as the association with health complications have raised concern to this disease 1. The diagnostic still presents failures, since the most used method worldwide, the wet mount examination, has low sensitivity. In addition, the report of positive cases for trichomoniasis is not mandatory and there is no vigilance system to detect the increasing antimicrobial resistance 2,3. To aggravate the scenario, there is no alternative treatment to the current Food and Drug Administration (FDA) approved drugs, the nitroimidazoles metronidazole (MTZ) and tinidazole (TNZ) 4. To achieve success in parasitism, the trichomonads pathogenesis against host cells is a complex process that includes dependent- and independent-contact mechanisms. Moreover, *T. vaginalis* is amitochondriate and presents a large genome with 176 Mbp distributed into six chromosomes, distinguishing features that make it a valuable cellular and molecular model 5.

Overall, excellent papers 6,7,8,9,10,11,12,13,14,15,16 have been published in the last 20 years to highlight the importance of *T. vaginalis* infection to human medicine. This article contributes to claim the attention of public health policies to control this STD.

## *TRICHOMONAS VAGINALIS* AND TRICHOMONIASIS: ETIOLOGY, TRANSMISSION, AND DIAGNOSTIC CONSIDERATIONS

The parasite *T. vaginalis* is the etiologic agent of trichomoniasis. The infection occurs in the female and male urogenital tract and humans are the only natural host for the parasite 15. The parasite exhibits a piriform or round shape, with four anterior flagella and a well developed undulating membrane that are responsible for the characteristic motility essential for direct diagnosis 6. *T. vaginalis* presents only the trophozoite stage, although, under stressful conditions, pseudocysts or endoflagellar forms have been described 17. The role of these resistant forms in the trichomonads life cycle is still not understood. In addition to its unique features, *T. vaginalis* presents hydrogenosomes instead of mitochondria, organelles that are involved in the metabolism adaptation to the hostile infection environment, including specific pathways of cell death 18,19,20.

The pathogen *T. vaginalis* is transmitted by sexual intercourse and the evidences that corroborate for the classification of trichomoniasis as STD are: (1) high frequency of infection in urethra and/or prostate of male partners of infected women; (2) the prevalence of infection is higher among female attending in STD clinics and among prostitutes than in postmenopausal women and virgins; and (3) the flagellates die outside of the human body, unless they are protected from desiccation 6. Studies that found *T. vaginalis* among young children contribute to maintain a high index of suspicion for sexual abuse 21,22. Although thought to be rare, the nonsexual transmission via fomites and possibly water has been described 23. The pathogen has also been isolated from the respiratory tract of infants 24 and adults 25,26. Undoubtedly, while producing a nuisance infection, *T. vaginalis* must be considered a clinical pathogen rather than commensal organism.

The trichomoniasis diagnosis must be laboratorial as the symptomatology could lead to confusion with other STDs. Accurate diagnostic procedures are essential to confirm trichomoniasis and direct to the appropriate treatment contributing to control the infection propagation 1. The most used method for diagnosis is the microscopic examination of wet mounts, which establishes the diagnosis by detecting actively motile organisms 4. Although this is the most practical and rapid method of diagnosis (allowing immediate treatment), it is relatively insensitive. Immunodiagnostic such as direct immunofluorescent antibody staining is more sensitive than wet mounts, but technically more complex 4. Serological ELISA has been reported to present higher sensitivity than microscopy with detection of trichomoniasis in asymptomatic population 27.

Two very sensitive tests approved by the FDA to detect *T. vaginalis* in vaginal secretions include the OSOM Trichomonas Rapid Test (Sekisui Diagnostics, Framingham, MA), an antigen-detection test using immunochromatographic capillary flow dipstick technology that can be performed at the point of care. The sensitivity and specificity for OSOM Test are 82%-95% and 97%-100%, respectively 28. The other test is the Affirm VP III (Becton Dickinson, Sparks, MD), a DNA hybridization probe test that evaluates for *T. vaginalis*, *Gardnerella vaginalis*, and *Candida albicans*, with sensitivity and specificity of 63% and 99.9%, respectively 29. Although very efficient, both tests are not cleared for use with specimens obtained from men 4.

As now updated by the STDs Treatment Guidelines from The Centers for Disease Control and Prevention (CDC, US) 4 the culture of the parasite is no longer considered the gold standard for diagnosing *T. vaginalis* infection once effective molecular detection methods are available. Culture has a sensitivity of 75%-96% and a specificity of up to 100%, but results are not available for 3 to 7 days. In women, examination should be performed on vaginal secretions. In men, anterior urethral or prostatic secretions should be examined, although urine can also be screened for *T. vaginalis* in both sexes and under nucleic acid amplification tests (NAATs). The APTIMA *T. vaginalis* assay (Hologic Gen-Probe, San Diego, CA) detects RNA by transcription-mediated amplification from vaginal, endocervical, or urine specimens from women with a clinical sensitivity of 95.3%-100% and specificity of 95.2%-100% 30.

In general, both guidelines from East European countries 31 and from CDC 4 recommend the following procedures for the laboratory diagnosis of trichomoniasis: (i) to perform diagnostic testing in all women with vaginal discharge, especially in high prevalence settings (e.g., STD clinics) and for asymptomatic persons at high risk for infection (e.g., persons with multiple sex partners, exchanging sex for payment, illicit drug use, or a history of STD); (ii) to employ NAATs or culture if no trichomonads are detected on microscopic examination of the wet mount preparation and there is a strong indication of infection. It is our understanding that highly sensitive (e.g., NAATs or culture) tests are not feasible in most laboratories especially from developing countries. In such cases, the wet mount examination of vaginal and urethral secretions and the urine sediment with careful specimen preservation and immediate microscopic examination can improve diagnostic sensitivity. Although *T. vaginalis* may be an incidental finding on a Papanicolaou test, neither conventional nor liquid-based Pap tests are considered diagnostic tests for trichomoniasis, because false negatives and false positives can occur 4. In addition, stained smears by Giemsa or Leishman at clinical settings are being discouraged 31.

## TRICHOMONIASIS IN NUMBERS

Trichomoniasis is the most common non-viral STD in the world. The WHO estimative performed in 2008 shows an incidence of 276 million new cases each year and a prevalence of 187 million of infected individuals with ages between 15 and 49 years-old 2. The incidence of infection depends on several factors including age, sexual activity, number of sexual partners, other STDs, menstrual phase, diagnosis techniques, social, and economic conditions. The prevalence is high among low social income patients from gynecologic and STDs clinics. The flagellates do not survive outside the human body unless they are protected from drying. Live *T. vaginalis* has been found in urine and in semen after several hours of exposure to air 6.

The worldwide prevalence of trichomoniasis is much higher than other curable STDs such as gonorrhea and syphilis, both counting for 36.4 million cases, and Chlamydia infection, with 100.4 million of infected adults. In the USA, several studies have determined the trichomoniasis prevalence in the range of 2.5% to 26.2% 32,33,34,35,36,37,38,39,40. Considering the Asian continent, consistent survey reports revealed prevalence values of 7.8% in South Korea 41 and 8.5% in India 42. Trichomoniasis prevalence varied from 8.4% to 48% among Indigenous patients in Australia 43,44. The Nordic countries in Europe account with 1.5% for the trichomoniasis prevalence 45 and in South Africa the prevalence was 6.5%, excluding co-infection cases with HIV 46. Studies in Latin America revealed similar prevalence values of 7.6% in Argentina 47 7.8% in Chile 48 and 9.1% in Peru 49. In Brazil, prevalence ranged from 2.6% to 20% among women 50,51,52,53 and the Health Ministry estimates a general prevalence of 15% 54. These uncertain data are due to the limitations in sample selection, since it is not representative of the Brazilian population in general.

In this context, Secor *et al*. 1 alert to the classification of trichomoniasis as neglected disease since the prevalence data are underestimated due to failures in diagnosis as consequence of insensitive methods or lack of testing in asymptomatic patients, and limited knowledge related to infection duration 55. Moreover, trichomoniasis is not notifiable, and there is no vigilance system to detect drug resistance, with low attention in the public health programs for STDs control 2,3. As a consequence of this overlooking, high costs and healthcare burden associated to trichomoniasis account to $24 million per year in the United States 56. The health complications caused by *T. vaginalis* aggravate the situation, as unrecognized costs with pregnancy adverse outcomes, infertility, cervical and prostate cancers are of concern. The estimated cost of the *T. vaginalis*-attributable HIV infections is approximately $167 million per year 57.

## THE TRICHOMONIASIS CLINICAL SPECTRUM AND HEALTH CONSEQUENCES

The *T. vaginalis* infection results in a variety of clinical manifestations - in most cases the patients are asymptomatic, but some may develop signs typically associated to the disease. Moreover, beyond the symptoms, the main issue concerning trichomoniasis is its relationship with serious health consequences such as cancer 58,59,60, adverse pregnancy outcomes 61,62,63,64, infertility 65,66, and HIV transmission and acquisition 67,68.

Studies have shown a wide divergence in the statistics on symptomatology of trichomoniasis. A couple of years ago, the infection was traditionally known as symptomatic in women and asymptomatic in men. The data of symptomatic women ranged between 50-75% 6,7,69 while in men the percentage was 15-50% 7,70. Currently the scenario is changing and recent data have mentioned that around 80% of *T. vaginalis* infections are asymptomatic in both men and women 16,32,71.

The preferential cells infected by the parasite are those of squamous epithelium. In women, the major infection site is the vagina but urethra and endocervix are also reached by the trophozoites 7,69,72. The normal vaginal pH is 4.5 and it is increased to 5 or more in presence of *T. vaginalis*. This enhancing of pH promotes the reduction of *Lactobacillus acidophilus* presence - the healthy microbiota which protects the vaginal epithelium - and consequently, contributes to the multiplication of anaerobic bacteria responsible for the bacterial vaginosis 6,12. This disturbance of genital tract site does not necessarily lead to a symptomatic condition. Although most of the literature establishes the incubation period of trichomoniasis as 4 to 28 days, this period is not clearly known yet 73, and one third of women become symptomatic within 6 months 6.

Among the symptomatic women, the main complaints are vaginal discharge, pruritus, odor, and irritation 72. The vaginal discharge is a classical signal of trichomoniasis and it is due to intense leukocytic infiltration within the genital tract as a result of the death of epithelial cells which promotes inflammation and leads to an increased number of polymorphonuclear leukocytes in vaginal fluid 74. The typical discharge is recognized as frothy and yellow/green; however, the aspect and consistency of it may be widely variable among the patients 70,73. Moreover, the vagina and cervix of women with trichomoniasis may be erythematous and edematous, and when punctuate hemorrhagic spots are found on the mucosa this condition is known as *colpitis macularis* or "strawberry cervix". This clinical sign is the most specific indicative of trichomoniasis although it is clinically diagnosed only in 2-5% of women 7. Some patients have still reported dysuria and lower abdominal pain. It is important to highlight that the infection symptoms are cyclic and more intense around the menses period because of the effect of iron on parasite pathogenesis 6. These clinical features may be associated with vaginitis, cervicitis and other complications. Endometritis, adnexitis, pyosalpinx, and atypical pelvic inflammatory disease are all disorders of the female genital tract related to *T. vaginalis* infection 55,75. Importantly, trichomoniasis may also impact on the pregnancy course, causing low birth weight, premature rupture of membranes and preterm delivery 64. There are some evidence that *T. vaginalis* infection can be transmitted vertically leading to cases of vaginal and respiratory infections in neonates; fortunately, the clinical improvement in these patients was reported after MTZ treatment or even with only supportive care 24. Another important issue regarding complications of trichomoniasis in women is its involvement with an increased risk of cervical cancer. Some studies have pointed *T. vaginalis* as a predictor for cervical neoplasia since there is a high relative risk of preinvasive lesion and invasive cancer in patients with trichomoniasis 60. A meta-analysis found that the parasite was associated with a 1.9-fold risk of this cancer 58.

The spectrum of trichomoniasis in men is less well characterized than in women once the infection is commonly self-limited and transient 12,73. These characteristics may be associated to the oxidative nature of male genital fluid that is hypothesized to be inhibitory to certain pathogenic factors as well as to the zinc concentration in prostatic fluid which acts as cytotoxic factor 66. However, *T. vaginalis* is a recognized cause of urethritis accompanied by scanty, clear to mucopurulent discharge, dysuria, and mild pruritus or burning sensation immediately after sexual intercourse 6. Other complications include prostatitis, balanoposthitis, epididymo-orchitis, and possibly infertility. There is not a consensus on the relationship between trichomoniasis and fertility, but in recent studies the parasite has been considered a contributing factor to male subfertility or infertility. As possible mechanisms involved in this case are the chronic infection, the cell lysis with toxicity to the sperm and the inflammatory process 66. *T. vaginalis* may be also related to cancer in men. To date, there are few studies investigating the association between the protozoan infection and prostate cancer risk 76,77,78. Although conflicting results have been found regarding *T. vaginalis* serostatus and prostate cancer, recent evidences strongly suggest this association 77,78. The frequent chronic course of the infection in men turns possible that the parasites ascend to the prostate and establish a site of inflammation that may lead to prostate cancer 79.

Certainly, one remarkable aspect in *T. vaginalis* infection is its positive association with both transmission and acquisition of HIV. The evidences that corroborate to this concern are substantial although still underappreciated 80,81,82. Studies have shown that trichomoniasis is associated with as much as a 2.7-fold increase in the risk of HIV acquisition 16. This data is especially significant taking into account the high prevalence of trichomoniasis within the general population, and in particular within risk groups 7. Some approaches (e.g., mathematical modeling) have been developed to estimate the number of transmitted HIV infections attributable to *T. vaginalis*, and the high efficacy of these methods are closely related to the need of improving the parasite diagnosis 68. The main discussed mechanisms by which *T. vaginalis* may enhance HIV acquisition are microhemorrhages in the mucosa caused by the flagellated, inflammatory response of vaginal, exocervix, and urethral epithelia followed by the recruitment of target immune cells, of secretory leukocyte protease inhibitor, and association with increased HIV viral load in genital secretions 68,82. Finally, the *T. vaginalis* control, through prevention, diagnosis and treatment, may have a pivotal impact on preventing HIV acquisition and transmission.

## PATHOGENICITY - OPENING THE "BLACK BOX" OF *TRICHOMONAS VAGINALIS* INFECTION

The *T. vaginalis* infection is very complex with a broad range of symptoms which may be attributed to distinct pathogenic process mediated by the parasite through contact-dependent and -independent mechanisms 8. The colonization of the infection site is initiated when the parasite triggers cellular damage in the host tissue by secreting a wide variety of molecules, known as cytolytic factors. *Trichomonas vaginalis* factor (TvF), a 250 kDa cytolytic effector, causes cell rounding and clumping without lysis 83. Another soluble factor released into the medium by the parasite in contact with cells is a glycoprotein with 200 kDa, known as cell-detaching factor (CDF) which promotes cell detachment 84. In *T. vaginalis*, high levels of proteolytic activity were attributed to cysteine proteases (CPs), proteins localized in parasite surface, although only a few CPs have been demonstrated and characterized 85. It has been shown that the parasite could modulate cell recognition and adhesion to the epithelial host cells through the proteolytic activity mediated by *T. vaginalis* CPs 86. In addition to the essential role for the colonization in the site of infection, these proteins play an important function in evading host immune defenses, since they degrade IgA and IgG antibodies as well as human extracellular matrix and complement proteins 87,88. The synthesis and proteolytic activity of certain CPs are modulated by environmental factors such as iron, pH, temperature, and polyamines 89.

Red blood cells are a main source of iron and lipids for *T. vaginalis* metabolism and erythrocytes lysis mediated by the parasite have already been demonstrated *in vivo*. It has already been suggested that haemolytic activity contributes to acquisition of nutrients, mainly iron, and these mechanism may be responsible for the exacerbation of symptoms observed during and following menstruation 90. Haemolysis is considered a complex process possibly involving several molecules as surface CPs, pore-forming proteins (PFPs), and phospholipase-A-like proteins, which have already been demonstrated as cytolytic factors in *T. vaginalis* 91,92,93,94,95. Haemolysis involves the regulation of temperature, concentration of Ca^++^ and pH (as acid environment is required) and the activity of PFPs 96. These PFPs contribute to cell lysis and death by forming transmembrane channels in the lipid membrane of target cells which leads to osmotic lysis 97.The presence of these PFPs have already been observed in other parasitic protists such as *Entamoeba histolytica* 98 and *Naegleria fowleri* 99 known as amebapores and naegleriapores, respectively. These PFPs are members of a conserved family of saposin-like proteins (SAPLIPs) that are found in phylogenetically distant organisms (e.g., protists and mammals). Several functions have been attributed to these proteins, however the interaction with lipids is a common hallmark attributed to this family. Twelve SAPLIP predicted genes (*TvSaplip1* and *12*) have been identified in *T. vaginalis* by genomic analysis. Based on the characteristics displayed by TvSaplips family, these predicted proteins named trichopores, are good candidates as effectors contributing to the cytolytic effects of *T. vaginalis*. Taking into account the heterogeneous nature of SAPLIP activities, it is not plausible to attribute to all TvSaplips the direct involvement in the cytopathogenicity of this parasite and other biological roles may be involved 100.

Recent studies demonstrated the secretion of exosome-like vesicles by the parasites beyond protein and soluble factors. Genetic studies identified tetraspanins, proteins that are markers of exosomes 101. *T. vaginalis* exosomes are about 50-100 nm in diameter and contain RNA and a variety of proteins. Remarkably, *T. vaginalis* exosomes demonstrated to bind to host cells and modulate parasite virulence against vaginal and prostate cells. Moreover, these vesicles demonstrated to have immunomodulatory properties, enhancing the possible role of the secreted molecules in the establishment of the infection 102.

Also important in modulating parasite-host cell interaction, another factor has been described, the *T. vaginalis* macrophage migration inhibitory factor (TvMIF) which is 47% similar to human macrophage migration inhibitory factor (HuMIF), a proinflammatory cytokine 103. It has been shown that TvMIF binds with high affinity to the human CD74 MIF receptor which activates cascades involved in cell proliferation and invasion. The presence of anti-TvMIF antibodies indicates that the factor is released by *T. vaginalis* and may result in inflammation and cell proliferation, thus activating pathways that contribute to the promotion and progression of prostate cancer 103.

The multifactorial nature of trichomonal pathogenesis also involves a sequence of events, where contact-dependent mechanisms play crucial roles. Upon contact with host cells, the parasite undergoes a drastic morphological shift. The free-swimming piriform trophozoites transform into an ameboid form leading to a tight association to the target cells 8. Actin proteins participate at this step inducing the cytoskeletal rearrangement and cellular proliferation. In this way, Gould *et al.* 104 showed the up-regulation of actin and actin-associated genes of *T. vaginalis* after contact with vaginal epithelial cells (VECs). While α-actinin is distributed throughout the cytoplasm when the cell is pear-shaped, the protein localizes only at the cell periphery when the trophozoites are in the ameboid form. The morphological transition from pear-shaped flagellates to tissue-feeding and actively dividing amoeboid organisms occurs in a few minutes and represents a crucial step of the infection process 10.

The typical mucous layer covering the VECs is part of the non-specific host defenses 105. The parasite can cross this barrier by binding and degrading mucin - a large glycoprotein with gel-like property that forms a lattice structure and serves as a formidable physical barrier to microbial invasion. *T. vaginalis* binds to mucin, possibly via lectin-like adhesion, and secretes mucinases able to degrade the protein over a pH range of 4.5-7.0 106. Directly related to those processes is cytoadherence - the major event of *T. vaginalis* pathogenesis. The mechanisms of cell adhesion are extensively studied in the parasite and up to now three major classes of molecules show evidence to be involved in the cytoadherence: lipophosphoglycan, adhesins and a collection of membrane proteins that have been recently identified through genomics and proteomics 87.

The *T. vaginalis* lipophosphoglycan (TvLPG) is one of the most abundant components of the glycocalyx - the outer layer of the cell membrane formed by different carbohydrate-associated molecules - that binds to galectin-1 and -3 receptors in the host cells 107,108. TvLPG plays a role in the parasite-host cell interaction to VECs once *T. vaginalis* mutant cells deficient in TvLPG glycosylation showed reduced adherence and cytotoxicity to human cervical cells 109. This molecule also participates in parasite virulence modulating inflammatory responses of epithelial cells and macrophages 110.

The second class of *T. vaginalis* proteins related to adherence comprises the named adhesins - five proteins (AP120, AP65, AP51, AP33, and AP23) that apart from AP23, are abundant metabolic enzymes primarily involved in carbohydrate metabolism and found in the hydrogenosome 111. Conversely, it has been already demonstrated that these proteins are also present on parasites surface 112,113, contributing to the hypothesis of their dual function: metabolic proteins and adhesins 114. This family of proteins also participates in the molecular mimicry mechanisms involved in immune evasion 8. Apart from the studies showing the surface localization of the adhesins, it has already been shown that these proteins are exclusively situated in the hydrogenosomes 115 and that they lack some features that are present in true adhesion proteins like transmembrane domains 115. The precise role of the adhesins in pathogenesis is still uncertain as some studies have already verified the interaction of these proteins with the host cell surface 116,117 in contrast with authors that demonstrated that *T. vaginalis* binds to cell target in the absence of membrane proteins 118. In agreement with the hypothesis that attributes a lack of adherence specificity for the adhesins, data demonstrated that AP51 and AP65 bind to haem and haemoglobin, a feature that evidences a function in the nutrient acquisition and metabolism, not related to adhesion properties 119. More studies, especially on the molecular basis are required in order to support that *T. vaginalis* adhesins acts as dual function proteins, verifying specific binding in cell targets as the recruitment of these proteins to the surface of the parasite.

Some studies have already demonstrated the regulation of adherence levels promoted by environmental regulation in the adhesins synthesis and metabolism. High levels of iron present in a complex supplemented medium lead to increased levels in trophozoites adherence 120. The increase was specially attributed to iron as parasites cultured in a low-iron medium and in the presence of salts other than iron were unresponsive to changes in adherence levels 120. Additionally, it was demonstrated that the higher adherence levels were a result of increased gene expression of AP65, AP51, AP33, and AP23 adhesins 120. Regarding the localization and compartmentalization of these proteins in *T. vaginalis* under contact with epithelial cells, it was already verified that high-iron-grown parasites co-expressed adhesins on the surface and intracellularly in contrast with low-iron parasites 121. More significantly, the study showed that MR100 trichomonads, a drug-resistant isolate lacking hydrogenosome proteins and adhesins, presented non-adherent profile 121. Besides iron modulation, *T. vaginalis* adhesion under contact with epithelial cells may also be regulated by other environmental mechanisms. When comparing the interaction of *T. vaginalis* adhesins with epithelial cells of fresh clinical and long-term-grown isolates it has been shown that fresh isolates presented greater amounts of adhesins, which corresponded to higher levels of cell adherence 117. These data suggest that some virulence factors that are still present in fresh clinical isolates may interact and regulate the adhesin metabolism and expression 117.

The last group of molecules speculated to be associated to the parasite adherence are the surface proteins, as BspA (*Bacteroides* surface protein A)-like. BspA-like are the largest surface protein family identified in *T. vaginalis* with evidence of expression for 721 members 104,122. Bacterial BspAs are able of mediating binding to host epithelial cells, extracellular matrix proteins and cell aggregation 8,122. Similarly, *T. vaginalis* BspA-like proteins are strong candidates of surface proteins mediating interaction with various mucosal hallmarks including: mucus, VEC, urethra epithelial cells, and vaginal microbiota 122. *In silico* analysis reveals other transmembrane proteins that are possibly involved in the host-parasite interaction, which comprise the GP63-like, subtilisin-like, serine proteases, and calpain-like cysteine proteases 115. However, although genomic and proteomic analyses have identified these proteins on the parasite surface, none of them have been characterized in detail and their putative role in host-parasite interactions is only hypothesized 10.

Another important factor contributing to *T. vaginalis* pathogenesis is the high cytotoxic potential of the parasite. Its ability to promote cytolysis followed by phagocytosis is what triggers the disruption of cell monolayers 8. Many are the factors involved in these processes, including contact-independent mechanisms. When attached to the parasite, the host cells may be phagocyted both by a ‘sinking’ process without any apparent participation of plasma membrane extensions as by the classical phagocytosis where pseudopodia are extended toward the target cell. Dramatic changes in the distribution of fibrillar actin have also been reported, which may facilitate the ameboid morphological transformation observed during phagocytosis 123. After the internalization of bacteria, yeasts and cells such as VECs, cervical and prostate cells, leucocytes and erythrocytes, the parasite digests the material in lysosomes 124. Hemolysis is another issue closely related to *T. vaginalis* cytotoxicity since the erythrocyte lysis is one source of important nutrients such as lipids and iron 90. This process is mainly contact-dependent and surface cysteine proteases, pore-forming proteins and phospholipase-A-like proteins are involved 93,125.

The host defense in response to *T. vaginalis* infection involves multiple mechanisms such as non-immunological factors, non-specific and specific mechanisms of the innate immune response 55,73. Non-immunological factors include the effects of environmental elements such as iron, zinc and polyamines, which directly modulate the expression of virulence genes in the parasite 8. In this sense, it was already shown that iron mediates *T. vaginalis* resistance to complement lysis due to proteinase degradation of C3 on the trichomonal surface 126.

The immune system of mucous layer is the first line of defense against pathogenic organisms in the urogenital tract and involves both innate and adaptive immune responses, including cellular and humoral immunity. Trichomoniasis does not produce an effective permanent immunity, which may lead to recurrent infection, consequently, innate immunity response has become crucial in the infection control 55. Upon contact with host cells and binding through LPG, *T. vaginalis* trophozoites trigger an inflammatory response in the VECs through the release of cytokines and chemokines, mainly interleukin-8 (IL-8), interleukin-6 (IL-6) and macrophage inflammatory protein (MIP-3α) 55. IL-8 production and release is also mediated by human neutrophils, major immune cells recruited to the site of inflammation and the predominant inflammatory cells found in the vaginal discharges of patients infected with *T. vaginalis* 127. Additional innate immune mediator induced by *T. vaginalis* is nitric oxide, which is produced by several cell types such as neutrophils and macrophages. It was already demonstrated that *T. vaginalis* trophozoites in contact with human neutrophils are able to stimulate the release of high levels of nitric oxide through the nitric oxide synthase 128.

Despite the predominance of innate immune responses, adaptive immunity mediated by the production of parasite-specific antibodies may play an important role in the infection control by host cells, since IgA and IgG immunoglobulins are detected in vaginal secretions of symptomatic women 129. In man, IgG1 and IgM antibodies detected may be involved in the establishment of symptomatic trichomoniasis, compared to asymptomatic cases 130. However, all antibodies produced and/or secreted during trichomoniasis only promote a limited protection to the parasite gradually declining after the eradication of infection in a period of six to twelve months. After infection, *T. vaginalis* specific antibodies and memory B cells are not found in the circulation, leaving the host without defense mechanisms against a possible reinfection 131. For this reason, it becomes so complex to establish the presence of antibodies in the diagnosis of trichomoniasis as well as to progress in research for effective vaccines.

Regardless of several innate and adaptive responses triggered by the host cells in order to control the infection, *T. vaginalis* evolved diverse immune evasion mechanisms. The secretion of proteases, specifically CP which degrades human immunoglobulins, not only keeps the survival of the parasite but also supplies nutritional demands trough hemolytic properties 125. Another important evasion mechanism comprises molecular mimicry in which parasite covers its membrane with molecules homologous to host proteins. The *T. vaginalis* adhesins (AP65, AP51 and AP33) are homologous to host metabolic enzymes and plasma proteins, in attempt to avoid the recognition by the host immune system 8. The secretion of immunogenic soluble proteins into the infection site by the parasite seems to neutralize circulating antibodies and facilitates the continuous colonization and infection of the vagina 7. Despite all the immune responses mediated by the host cells, *T. vaginalis* is able to evade those mechanisms and displays the whole pathogenic potential which turns trichomoniasis a chronic and persistent infection.

Finally, *T. vaginalis* may exert a "Trojan horse" role in the microbial environment, since it can have a symbiosis relationship with *Mycoplasma hominis*, a small bacterium associated with urogenital and respiratory system infections 132. Studies demonstrated that the association of both microorganisms presents prevalence values greatly ranging from 20 to 92% 132,133,134,135 and it might influence the cytopathogenic effect of *T. vaginalis* on epithelial cells and inflammatory responses 136,137. In addition, a strong relationship between *M. hominis* co-infection and MTZ resistance *in vitro* was shown 135 contrasting to studies that revealed the lack of this correlation 133,134.

Besides *M. hominis*, *T. vaginalis* can also be infected with four viruses, known as *T. vaginalis* virus (TVVs) that are members of the Totiviridae family 138. Large variability is found in the prevalence values, from 13 to 90% of *T. vaginalis* isolates harboring TVVs 134,139,140. The participation of the virus in the virulence of *T. vaginalis* is under investigation. An association between the presence of viruses and expression of immunogenic proteins on the trichomonal surface, variations in protozoan phenotypes, and upregulation of certain virulence factors has been shown 141. Notably, Fichorova *et al*. 142 suggest focus in TVVs as targets for new therapeutic paradigms thus preventing the inflammatory sequelae caused by virus-harboring parasites.

Efforts have been made to know how *T. vaginalis* succeeds parasitism and infection, and the studies on genomic, proteomic and transcriptomic have brought considerable advances on the information on gene and protein expression that contributes to the comprehension of several biological functions 143. One of the cornerstones that partially explain the parasite complexity is the extensive gene duplication and presence of multiple gene families in the *T. vaginalis* genome as well as the impressive percentage of 86% hypothetical proteins 5,143. The publication of the first *T. vaginalis* genome in 2007 resulted in considerable advances in the knowledge of the biology of the parasite and continuous efforts on the "omics" database, TrichDB, are being stimulated to contribute to solve the remaining gaps in the field. Huang *et al*. 144 constructed a proteome reference map of *T. vaginalis* by using two-dimensional electrophoresis combined with matrix-assisted laser desorption ionization time-of-flight mass spectrometry analysis and found that proteins related to carbohydrate metabolism represented the most abundant category in the *T. vaginalis* trophozoite stage 144. Following this initial analysis, several recent reports at the transcriptomic level have demonstrated the parasite responses to stress conditions such as glucose and iron restrictions by using next generation RNA sequencing 145,146. Glucose restriction elicits trichomonads antioxidant ability and autophagy to maintain survival trough a metabolic reprogramming. In the same way, nitric oxide exerts a cytoprotective effect on iron-deficient *T. vaginalis* by maintaining the hydrogenosomal membrane potential 146. Furthermore, the transcription of iron-regulated and iron-independent gene copies was analyzed and multiple gene copies were shown to be advantageous for the parasite to differentially express genes and proteins under stringent regulation in variable environmental conditions 147. Besides nutrients metabolism, functional analysis showed the effects of cold temperature on cellular pathways including H_2_O_2_ tolerance, activation of the ubiquitin-proteasome system, induction of iron-sulfur cluster assembly, and reduced energy metabolism and enzyme expression 148. Moreover, considering the crucial pathogenic process of cytoadherence, integrated transcriptomic and proteomic approaches revealed that cysteine peptidase, glyceraldehyde-3-phosphate dehydrogenase, and stress-related proteins were upregulated in the fibronectin-adherent parasites, indicating that these genes and proteins may play critical roles in the response to adherence 149. Another approach to investigate protein expression was the phosphoproteome involved in the morphological alterations from the pear-shape form to ameboid and pseudocysts in *T. vaginalis*, where a total of 93 phosphopeptides originating from 82 unique proteins involved in these processes were found 150. Next-generation sequencing-based RNA sequencing was also employed to analyze the transcriptome of *T. vaginalis* in response to tetracycline, a broad-spectrum antibiotic with activity against several protozoan parasites 151. Tetracycline was cytotoxic against MTZ-sensitive and -resistant *T. vaginalis* isolates, inducing some features resembling apoptosis, altering the transcriptome via aminoacyl-tRNA synthetases and carbohydrate metabolism, and causing disruption on the hydrogenosomal membrane potential and antioxidant system. Altogether, these data revealed the potential of tetracycline as alternative therapeutic choice for treating MTZ-resistant *T. vaginalis* 151.

Overall, this intriguing extracellular pathogen establishes infection through coordinated crucial steps: morphological alteration from pear-shaped to ameboid forms followed by cytoadherence and release of virulence factors. This complex mechanism leads to tissue colonization with immune evasion, culminating in a good parasitism success. Although these pathogenic mechanisms have been progressively revealed, the continue efforts to elucidate the "trichomonads black box" are required.

## TREATING TRICHOMONIASIS: ARE WE SUCCEEDING?

Considering the whole spectrum of clinical manifestations and the complications arising from the infection, *T. vaginalis* vaginitis requires prompt and effective treatment. Nitroimidazole drug family, mainly represented by MTZ and TNZ, has been used as antitrichomonal agents for more than 30 years, being MTZ the treatment of choice 13. This class of drugs is the only one approved by the FDA for *T. vaginalis* infection treatment. These medications are widely available in public health systems and quite inexpensive, especially MTZ. TNZ has a longer half-life and reaches a higher genitourinary tract drug level than MTZ, but it is more expensive 9. Therapeutic approaches used in the treatment of trichomoniasis are local intravaginal applications, systemic oral medication and the association of both. There is also evidence that a spontaneous cure rate in the order of 20-25% is achieved 152. As *T. vaginalis* in women frequently infects the urethra and paraurethral glands cure and local medication reaches just around 50%, the oral medication treatment is preferred. A Cochrane review described that in most trials single dose treatment with any nitroimidazole drug leaded in trichomonicidal actions upon 90%. Despite rarely severe, side effects appeared to be relatively common and dose related 152.

According to the 2015 STD Treatment Guidelines from CDC the recommended regimens for treating trichomoniasis correspond to 2 g MTZ or TNZ orally in a single dose 4. MTZ gel is considered less efficacious than oral treatment (fewer than 50%) since topical preparations cannot achieve therapeutic levels in the urethra or perivaginal glands. As an alternative regimen, 500 mg oral dosage of MTZ can be used twice a day for 7 days 4. Distinct recommendation is given by the United Kingdom on the Management of *Trichomonas vaginalis* 2014, where TNZ 2 g orally in a single dose is considered an alternative regimen and the recommended regimen is based on the two possible doses of MTZ treatment 153. In order to compare the efficacy of different regimens of MTZ and TNZ some studies were already conducted. MTZ in two different single doses (1.5 or 2.0 g) demonstrated equivalent efficacy for trichomoniasis treatment 154. The multidose regimen (500 mg twice a day for 7 days) was more effective than the single dose (2 g orally) for the treatment of trichomoniasis among co-infected HIV-*T. vaginalis* subjects 155. It is important to emphasize that this study was the first to evaluate the effectiveness of treatment for trichomoniasis among HIV-infected women. These data suggest that the recommended standard regimen of MTZ may need to be reconsidered for HIV-infected women reinforcing that more studies are necessary to investigate optimal treatment regimens for distinct patient populations presenting co-infecting pathogens. A different approach was evaluated using single-dose intravaginal MTZ (2 g) in comparison to single-dose oral MTZ (2 g) which demonstrated that the intravaginal use was inferior to single-dose oral MTZ, failing as an alternative therapy 156. Several other antimicrobial preparations, mostly used for bacterial vaginosis treatment are also used for *T. vaginalis* infection, although with lower effectiveness than MTZ 4. The overall cure rates are not significantly different between MTZ and TNZ regimens and no significant differences in adverse events across treatment were obtained 152,157. The side effects are also a disadvantage for the treatment with MTZ or TNZ.

In relation to possible side effects during treatment, patients should be recommended not to ingest alcohol for at least 48 to 72 hours due to possible toxicity effects. Referring to allergies, hypersensitivity reactions have been described in patients using both MTZ and TNZ and it is unknown whether there is cross-reactivity between the two agents 152. Considering that nitroimidazoles are the only therapeutic option available, it is important to take an accurate history to establish that a true allergy exists otherwise standard treatment will be unviable. Furthermore it is not well established if TNZ is well tolerated in a patient with MTZ allergy. Adverse reactions which may occur include anaphylaxis, skin rashes, pustular eruptions, pruritus, flushing, urticaria, and fever 158.

Vaginal trichomoniasis has been associated with adverse pregnancy outcomes, particularly preterm delivery and low birth weight 61. Multiple studies and meta analyses have not demonstrated an association between MTZ use during pregnancy and teratogenic or mutagenic effects in newborns and infants 159,160,161. Symptomatic pregnant women should be treated at diagnosis, although some clinicians prefer to delay treatment to the second trimester. The safety of TNZ in pregnant women, however, has not been well established. In lactating women who are administered MTZ, avoiding breastfeeding during treatment and for 12-24 h after the last dose will diminish the exposure to MTZ. For women treated with TNZ, interruption of breastfeeding is recommended during treatment and for 3 days after the last dose 152,162.

The reliance on a single therapeutic class is problematic since resistance to nitroimidazoles is becoming widespread in *T. vaginalis* isolates. There is very limited information on the prevalence of resistance to MTZ among clinical isolates of *T. vaginalis*, especially because no surveillance systems are implemented to detect treatment failures due to resistance and antibiotic susceptibility testing for *T. vaginalis* is not standardized. Studies indicate an increasing prevalence of 2.5 to 9.6% of MTZ-resistant isolates 3,163,164. Although the low prevalence of nitroimidazoles resistance occurs, more studies focused on this research area are urgent, as only two agents are available for treatment. Recent works are exploring genomic sequences aiming to identify possible target candidate genes in *T. vaginalis* drug resistance based on the role of these sequences in other organisms 143. It has already been shown that *T. vaginalis* presents homologs of bacterial nitroreductases and nitroimidazole reductases that are lacking in the majority of eukaryotes are related with reduced susceptibility to MTZ in *Helicobacter pylori* and *Bacteroides* 165. It is not still clear if these genes are associated with nitroimidazole sensitivity in the parasite, but it was demonstrated that these enzymes might activate MTZ in cytosol and hydrogenosome, opposing to previous reports of activation occurring exclusively by the hydrogenosomal enzymes pyruvate ferredoxin oxidoreductase and hydrogenase 166. More recently, 167 it was demonstrated the down-regulation or even absent activities of flavin reductase and alcohol dehydrogenase in *T. vaginalis* strains with high levels of MTZ resistance while thioredoxin activity was nearly equal in all strains evaluated, conflicting with previous data that indicated the contribution of these enzymes in resistance 168,169. It is important to emphasize that clinical resistance to MTZ in *T. vaginalis*, also known as aerobic resistance, is fundamentally different from high-level MTZ resistance induced in the laboratory, named anaerobic resistance 143. The anaerobic resistance is induced in the absence of oxygen and is a consequence of a loss of drug activating enzymatic pathways which are responsible for the reducing of the prodrug MTZ to toxic intermediates 166. On the other hand, aerobic MTZ resistance seems to be related to elevated intracellular oxygen concentrations in consequence of diminished oxygen scavenging capacity which interferes in the activation of nitroimidazoles 170. Taking into account the distinct profile observed in *T. vaginalis* isolates the development novel assay methods for detection and the identification of molecular mechanisms of resistance in the parasite are urgent 143.

Considering the possible failures during MTZ treatment either by adverse reactions or by the emergence of resistant clinical isolates, the development of an alternative treatment is recommended. The search for antiparasitic drugs has focused on the identification of active natural products from plant and marine microorganism extracts and compounds with promising anti-*T. vaginalis* activity (read more in Vieira *et al.* 171). The characterization of parasite biochemical and molecular targets such as flavin reductase 1, pyruvate-ferredoxin oxidoreductase, ferredoxin, and nitroreductases 172,173 is also a potential strategy for new therapeutics. In addition, the development of topic adjuvant treatments and the strategy of repositioning available compounds are included in the pharmacological approaches to expand the trichomoniasis treatment repertoire.

Another research area on new treatment for trichomoniasis and vaginal infections may be focused in to repurpose compounds for use in a new therapeutic application and on revisited drugs, which use has been discontinued for one specific approach but may be effective in another pathogenic context. In the first scenario, Goodhew and Secor 174, screened for 1040 drugs of the US Drug Collection Library for activity against susceptible and resistant *T. vaginalis* isolates. The study shows that among all those drugs no one was as effective as any of the 5-nitroimidazole drugs reinforcing the limitation in developing new therapeutic alternatives for the current therapy. Still of concern is the repurposing of miltefosine, a synthetic lipid analogue used for the treatment of cutaneous metastasis from mammary carcinomas and oral treatment of visceral leishmaniasis has already demonstrated anti-*T. vaginalis* activity in susceptible and resistant isolates 175,176. Other promising candidate is pentamycine, a macrolide antibiotic used for fungal and bacterial vaginitis with high activity against *T. vaginalis*. The effect is prompt and independent of under-lying MTZ resistance 177.

Although effective clinical treatment is widely available, *T. vaginalis* infection remains one of the most common STDs which answers the initial question - no, we are not succeeding in treating trichomoniasis. Reinfection by partners appears to be a major problem, especially when typical symptoms of the infection are absent. According to the most important guidelines, sexual partners should be treated simultaneously. Patients should be advised to abstain from sex at least one week and until they and their partner(s) have completed treatment and patient and partners are asymptomatic.

## STRATEGIES TO PREVENT OR CONTROL TRICHOMONIASIS

Strategies for trichomoniasis protection including sexual behavior, condom usage, and therapy have not contributed to the decrease on disease prevalence, pointing to the need for novel innovative approaches for protection. The *T. vaginalis* infection is curable but is currently far away to be controlled. The treatment with MTZ or TNZ is recommended by the CDC; however, cure failures remain problematic due to noncompliance, reinfection and/or lack of treatment of sexual partners, or inaccurate diagnosis since symptoms resemble other STDs. Moreover, increasing numbers of *T. vaginalis* isolates resistant to MTZ argue in favor to improve prevention tools and new treatment alternatives 3,164. Multipurpose prevention technologies are new, all-in-one tools being developed to protect against HIV, other STDs, and unintended pregnancy that, once validated, will certainly contribute to the control of these infections in the future 178.

Vaccine development has been hampered by the lack of long-lasting humoral immunity associated to the absence of good animal models 11. Only two candidates for trichomonal vaccine have been submitted in clinical trials in the last 50 years, with no success 179,180. Recently, Smith and Garber 181 have tested a FDA approved adjuvant, Alhydrogel, formulated with live, whole cell *T. vaginalis* in the mouse immunized model, with potential applicability. Among the animals tested as *in vivo* model for *T. vaginalis* infection: mouse, rat, hamster, guinea pig, rhesus monkey (*Macaca mulatta*), crab-eating macaque (*Macaca irus*), stump-tailed macaque (*Macaca arctoides*), pigtailed macaque (*Macaca nemestrina*), and squirrel monkey (*Saimiri sciureus*), the pigtailed macaque is the most promising model since it naturally harbors lactobacilli, has a vaginal pH of 5.5-8.0, sustains infection up to 2 weeks and responds to MTZ treatment 182,183.

A great challenge in trichomoniasis control resides in novel vaccine development associated to effective prevention tools. The goal of a vaccine is hard to be achieved due to intrinsic difficulties related to the multifactorial parasite pathogenesis and new alternatives for the treatment are also urgently needed.

## CONCLUDING REMARKS AND PERSPECTIVES

Despite all accurate studies that have been conducted to understand *Trichomonas vaginalis* and trichomoniasis, there is still a lot of knowledge hidden by this audacious extracellular pathogen. Our answer to the title is no, we are not giving the deserved attention to the most common non-viral STD in the world. Why? Probably because the infection does not directly cause death and health professionals in general are not aware about the serious consequences of the disease. Ideally a collaborative effort of researchers focused in studies on the *T. vaginalis* biology and pathogenesis, the improvement on diagnosis methods and detection of drug resistance in parallel with new treatment and prevention options are required to achieve the goal of reduction of *T. vaginalis* burden in humans.
